# Health benefits and harms of mammography screening in older women (75+ years)—a systematic review

**DOI:** 10.1038/s41416-023-02504-7

**Published:** 2023-11-29

**Authors:** Erin Mathieu, Naomi Noguchi, Tong Li, Alexandra L. Barratt, Jolyn K. Hersch, Geertruida H. De Bock, Elizabeth J. Wylie, Nehmat Houssami

**Affiliations:** 1 Sydney School of Public Health, The University of Sydney, Sydney, NSW Australia; 2 The Daffodil Centre, The University of Sydney, A Joint Venture with Cancer Council NSW, Sydney, NSW Australia; 3 Wiser Healthcare, The University of Sydney, Sydney, NSW Australia; 4 Department of Epidemiology, University of Groningen, University Medical Center Groningen, Groningen, The Netherlands; 5 BreastScreen Western Australia, Women and Newborn Health Service, Perth, WA Australia

**Keywords:** Breast cancer, Population screening

## Abstract

**Background:**

There is little evidence on the balance between potential benefits and harms of mammography screening in women 75 years and older. The aim of this systematic review was to synthesise the evidence on the outcomes of mammography screening in women aged 75 years and older.

**Methods:**

A systematic review of mammography screening studies in women aged 75 years and over.

**Results:**

Thirty-six studies were included in this review: 27 observational studies and 9 modelling studies. Many of the included studies used no or uninformative comparison groups resulting in a potential bias towards the benefits of screening. Despite this, there was mixed evidence about the benefits and harms of continuing mammography screening beyond the age of 75 years. Some studies showed a beneficial effect on breast cancer mortality, and other studies showed no effect on mortality. Some studies showed some harms (false positive tests and recalls) being comparable to those in younger age-groups, with other studies showing increase in false positive screens and biopsies in older age-group. Although reported in fewer studies, there was consistent evidence of increased overdiagnosis in older age-groups.

**Conclusion:**

There is limited evidence available to make a recommendation for/against continuing breast screening beyond the age of 75 years. Future studies should use more informative comparisons and should estimate overdiagnosis given potentially substantial harm in this age-group due to competing causes of death.

This review was prospectively registered with PROSPERO (CRD42020203131).

## Background

The majority of population-based organised breast screening programmes invite women aged 50-69 years to participate in mammography screening. This is based on evidence from randomised controlled trials (RCTs) which show that detection of breast cancer at an early-stage through mammography screening leads to a reduction in breast cancer mortality in this age-group [1]. Evidence for screening women 70–74 years comes mainly from observational and modelling studies [2, 3] and there is limited additional evidence from the RCTs, with some programmes expanding the target age-group for screening to include women up to 74 years of age [4]. For women older than 74 years, there is no trial evidence for the benefits and hence no specific guidance regarding the net health benefits (versus harms) of continuing mammography screening beyond that upper age limit of 74 years. Despite this, data indicate that screening women into their late 70s, 80s and 90s is occurring in practice [5]. In the US, where there is no stipulated upper age limit for breast screening, there are no clear recommendations as to whether to continue or stop mammography screening beyond 75 years of age [6–8]. The European Commission’s recommendations are for biennial screening in women aged 50–69 years and a suggestion of every 2–3 year screening up to age 74 years [9].

Australia actively recruits women aged 50–74 years for two-yearly (biennial) mammography screening for breast cancer. Prior to 2013–14, the target age-group included women aged 50–69, however this was extended to invite women aged 70–74, based on a recommendation in a programme evaluation report [10]. This report also recommended that women aged 75 years and over should no longer be eligible to attend the programme given limited evidence of health benefits. Nonetheless, currently, women aged 75 years and older can self-select to attend mammography screening. In 2016–2017, 14.4% of women aged 75–79 years, 5.4% of women aged 80-84 years and 1.2% of women aged 85 years and older underwent a screening mammogram despite not being actively invited to screening [11].

Globally, there is no consensus or uniform policy on whether mammography screening should be ceased or even discouraged in older women (and if so what upper age limit should be set) [12]. Importantly, there is little evidence on the balance between potential benefits and harms in older women (specifically 75+) in whom competing causes of death and co-morbidities could render routine screening relatively harmful, and a shorter life expectancy could reduce the likelihood of experiencing benefit from screening. To support population-level, as well as individual, decisions about the age or age-range to stop (or recommend against) screening, we conducted a systematic review of the evidence on the outcomes of mammography screening in older women.

The aim of this study was to systematically review and synthesise the evidence on the outcomes of mammography screening in women aged 75 years and older, to guide screening recommendations.

## Methods

We report our methods and results in line with the Preferred Reporting Standards for Systematic Reviews and Meta-analyses (PRISMA) and provide a completed PRISMA checklist (Supplementary Appendix 1).

### Search strategy

Our search strategy was developed based on a Cochrane systematic review of mammography screening (2013), with limits in place regarding age and publication date (Appendix 1). We searched three major databases (Ovid Medline, Embase and Cinahl), as well as hand searching all identified systematic reviews of breast cancer screening. We performed forward and backward citation tracking of identified relevant articles and contacted experts in the field for additional studies not located as part of the comprehensive search.

Searches were carried out in the specified databases for publications from 1990 to July 2022, with no language or other restrictions.

### Selection criteria

Studies that reported relevant outcomes data for women aged 75+ years undergoing mammography screening using any method (i.e. film, digital, tomosynthesis) in a comparative context (relative to another group; continuing beyond 74 vs stopping at younger age; or screening beyond 74 vs not screened) or a non-comparative design were eligible for inclusion in this review (case reports and case series were excluded). All studies that included women aged 75 years and over, were included, however some were later excluded at data extraction stage if data was not stratified to enable extraction for this age group.

Relevant outcomes included both the benefits (or surrogate outcomes from which benefits could be inferred) and harms of mammography screening. Such outcomes included: breast cancer mortality; all-cause mortality; incidence of advanced breast cancer (or breast cancer stage distributions); prognostic characteristics of screen-detected breast cancers; evidence on treatment patterns (including data on treatment-related morbidity, such as physical adverse effects of treatment, quality of life measures); false-positive mammography; overdiagnosis; overtreatment; anxiety or adverse impact on quality of life; false positive biopsy or surgery for benign findings, false-negative findings (reported as interval cancer rates).

### Study selection

Titles and abstracts were screened by one of three investigators (EM, NN, JH) and a research assistant for possible inclusion. Papers were excluded based on title/abstract if it was apparent that any of the inclusion criteria were not met. A random sample of 10% of titles and abstracts were double screened to ensure high levels of agreement. Any article which the investigator was unsure about was included in the list of full text articles to review in the second stage.

Full text versions of articles selected in the screening stage were reviewed independently by two of the four investigators confirm eligibility for inclusion. Disagreements at the full text stage were resolved by consensus.

### Data extraction

Data was extracted by two investigators independently (EM, NN, TL). After completion of data extraction, the two authors reviewed both sets of extracted data and checked for errors and disagreements. Any disagreements were resolved by consensus.

For studies with data not specifically separated into 75 years and older, email contact was attempted with the author requesting additional (age-group specific) data if available (up to two attempts).

### Data synthesis

Data from all included studies was extracted and synthesised through tabulation and a narrative synthesis was undertaken.

### Quality appraisal

Study quality was appraised by one investigator (EM) using the Risk of Bias in Non-randomised studies of interventions (ROBINS-I) tool [13] or the Quality of primary diagnostic accuracy studies (QUADAS-2) tool [14] with specific consideration of screening specific biases such as lead time and length time bias for observational studies. We also adapted the Risk of Bias criteria used by Carter et al. [15] for modelling studies (using two criteria: transparent assumptions; data validation). Other investigators (NH, JH) were consulted if there were any areas of uncertainty regarding the assessment of the risk of bias for included studies (see also Table 1).

**Table 1:** Summary of included studies.

Study Reference	Country	Participant population/age of women included	Study design	Screening context/setting	Screening process/screening interval	Comparison made	Total numbers in comparison groups	Follow-up/collection period	Risk of Bias^a^
Advani [16]	USA	66–94 years	Cohort study	Women who underwent screening mammography in 1999–2010 in a Medicare-linked BCSC database. Outcomes ascertained via linkage with regional SEER programmes or state tumour registries	At least one screening mammogram	66–74 vs 75–84 vs 85–94	304,334 vs 190,180 vs 30,346	Follow-up: Within one year or until next screen Collection period: 1999–2010	Serious
Arleo [17]	USA	Using Cancer Intervention and Surveillance Modelling Network (CISNET) to develop models	Modelling study	Using CISNET models to simulate and compare three screening mammography recommendations	Annual and biennial 75–79 years and 75–84 years	Different screening recommendations	1000 women screened	N/A	Low
Bennett [18]	England	All women self-referred to NHS screening programme 71 years and older	Screening evaluation study	Analysed data for all women in England over the age of 70 who self-referred in the 2-year period 2005–2008.	All women self-referred to NHS screening programme and attended one screen during the collection period	70–74 vs 75 and older	71–74: 86,743, 75+: 53,167	No follow-up Collection period:3 years (2005–2008)	Serious
Boer [19]	The Netherlands	MISCAN (Micro Simulation Screening Analysis) model starts with women aged 40, but paper focuses on 70 years and older	Modelling study	The MISCAN model (simulated model with 2-yearly screening carried out during a period of 27 years)	2-year interval	Optimistic variant vs pessimistic variant and 69 vs 99	Assuming 100% attendance rate; and realistic attendance and invitation starting at 51 years	N/A	High
Braithwaite [21]	USA	66–89 years	Cohort study	Data linkage, registries, women receiving (free) mammograms between 1999 and 2006	Annual (9–18 months) or biennial (>18–30 months)	66–74 vs 75–89	2993 women aged ≥66 and with breast cancer and 137949 women aged ≥66 and without breast cancer	Follow Up: Not stated, max of 7 years (1999–2006) Collection period: 1999–2006	Serious
Braithwaite [20]	USA	66–99 years	Cross-sectional study	Not reported	At least one screening mammogram	66–74 vs 75–84 vs 85-99	6587 (1.2%) were followed by biopsy within 90 days among 537254 screens (171,636 women).	No follow-up Collection period:1999–2010	Critical
Cate [22]	USA	75 years and older	Cross-sectional descriptive	Screening mammography	Not reported	No comparison	2057 in total	No follow-up Collection period:2013–2014	Critical
Demb [23]	USA	66–94 years	Cohort study	No less than 1 screening mammogram between 66 and 94 years	All women self-referred to screening NHS programme	66–74 vs 75–84 vs 85–94	222,088 in total	Follow-up: Median of 107 months (IQR = 65–120); Collection period: 1999–2010	Serious
Destounis [24]	USA	75 years and older	Cross-sectional descriptive	Data from 763,256 screening mammograms at Elizabeth Wende Breast Care (all ages)	Mammography but no further details.	No comparison	76,885 screening participants aged 75+	No follow-up Collection period: 10 years 2007–2017	Critical
El-Zaemey [4]	Australia	65 years and older	Cohort study	Free screening for all women ≥40 years offered every 2 years	All women aged up to 75 years are invited to participate in biennial screening programme. Women age 75years and older are self-referred.	65–69 vs 70–74 vs 75 and older	39,886 vs 26,432 vs 8763 (number of screens)	Follow-up: Up to 3 years Collection period: 2015–2017	Serious
Erbas [25]	Australia	40 years and older	Cross-sectional study	Free screening for all women ≥40 years offered every 2 years	A second or subsequent screen in a woman with at least one prior negative screen within the biennial programme.	40–49 vs 50–69 vs 70–74 vs 75 and older	27,661 vs 506,117 vs 87,545 vs 24,699 (646,022 in total)	No follow-up Collection period: 1993–2000	Serious
Garcia-Albaniz 2020	USA	70–84 years	Cohort study	20% of Medicare fee for service beneficiaries. No history of BC, continuous enrolment in Medicare Parts A and B for 12+ months	Stop screening (women who do not have any screening after baseline), Continue screening (women who continue annual screening (with 3 mth grace period)	Continue vs stop screening	1,403,735 (aged 75–84)	Follow-up: Median of 16 months (IQR = 14–32); Collection period: 1999–2008	Serious
Hartman [27]	USA	75 years and older	Cross-sectional descriptive	All screening mammograms performed at institution from 2007 to 2013, outcome is screen detected cancers at institution, free screening for all women annually	Annual breast cancer screening	No comparison	4424 in total	No follow-up Collection period: 2 February 2007–31 December 2013	Critical
Jansen [28]	The Netherlands	A computer model for the simulation of breast cancer screening (MBS) to calculate results of screening in terms of lifetime ≥20 years	Modelling study	The MBS model (simulated model with a stable Swedish population of one million women)	Annual mammography with single view	No comparison	One million	Lifetime (simulated)	Moderate
Jansen [29]	Sweden	A computer model for the simulation of breast cancer screening (MBS) to calculate results of screening in terms of lifetime ≥20 years	Modelling study	Using Swedish two county study and the MBS model	Annual mammography with single view	No comparison	One million	Lifetime (simulated)	Moderate
Kregting [30]	The Netherlands	40-84 years	Modelling study	920 breast screening strategies with varying starting ages (40–60) and stopping ages (64–84)	Screening intervals of 1-4 years	Many - have chosen annual screening 40-75 to annual screening 40-84	N/A	N/A	Moderate
Lansdorp-Vogelaar [31]	USA	US cohorts 66–90 years in 2010 with average health or one of four comorbidity levels - none, mild, moderate, or severe. Using models from CISNET	Modelling study	Simulated data. Assuming all undergo regular screening starting at 50 with biennial mammography and follow individuals for their remaining lifetime. MISCAN-Fadia (Microsimulation screening analysis – Fatal diameter) model and a G-E (Georgetown-Einstein) model	Regular screening starting at age 50 with biennial mammography	74 vs 76	Not reported	Lifetime (simulated)	Low
Malmgren [32]	USA	All women with primary breast cancer aged 50+ (75–94 years)	Cohort study	Cancer detected between 1990 and 2008, presenting at clinic. All women either patient, physician or mammography detected	Mammography but no further details	Mammography detected v physician detected v patient detected	5595 in total, 75+ = 950	Follow-up: not stated Collection period:1990–2008	Serious
Malmgren [33]	USA	All women with primary breast cancer aged 50+ (75–94 years)	Cohort study	Cancer detected between 1990–2011, presenting at clinic (using registry database). All women either patient, physician or mammography detected	Mammography but no further details	Mammography detected v physician detected v patient detected	1162	Follow-up: Mean 7.9 years (range 1.8–21 years) Collection period: 1990–2011	Serious
Mandelblatt [34]	USA	Using Cancer Intervention and Surveillance Modelling Network (CISNET) to simulate six models for women born in 1960 beginning at 25 yrs old	Modelling study	Using CISNET models to estimate the benefits and harms of alternate screening strategies	10 strategies each evaluated using annual and biennial schedule—a total of 20 strategies	Stopping screening	1000 women screened	N/A	Moderate
McCarthy [35]	USA	All women in the SEER database wo received a diagnosis of incident breast cancer in 1995 or 1996 and were linked with Medicare data. Aged 69 years and older	Cohort study	Cancer detected between 1987 and 1993 linked to Medicare records which indicated pattern of screening	Non-user (none in past 2 years), single user (once in the last 2 years), regular user (at least 2 screening mammograms at least 11 months apart in past 2 years)	i) Age at diagnosis ii) Non-users v regular users	67–74 *n* = 4609 75–84 *n* = 4072 85+ *n* = 1086 Screen-usage 67-74: Reg 29%; Non 18% 75–84: Reg 23%; Non 21% 85+: Reg 10%; Non 33%	Follow-up: 1–8 years Collection period: 1987–1993	Serious
McPherson [36]	USA	Caucasian women aged 65–101 diagnosed with invasive breast cancer from 1986 to 1994	Cohort study	Cancer detected between 1986 and 1994 identified from records in the Upper Midwest Oncology Registry System. Method of detection extracted	Mammography but no further details	Mammography detected v clinically detected cancers	75–79: 599 v 546; 80–84: 473 v 313; 85+: 451 v 148	Follow-up: not stated, but reports 8–9 years post diagnosis data Collection period: 1986–1994	Serious
Park [37]	USA	40–85 years at baseline	Cohort study	Women enroled in the California Teachers study (1995–1996) followed up with data linkage up until Dec 31 2015	Routine screening	Never/less frequent v biennial v annual screening	8024 women aged 75-85 at baseline	Follow-up: 20 years Collection period: 1995–2015	Serious
Randolph [38]	USA	All women in the SEER database wo received a diagnosis of incident breast cancer in 1995 or 1996 and were linked with Medicare data. Aged 69 years and older	Cross-sectional study	Cancer detected between 1995 and 1996 linked to Medicare records which indicated pattern of screening	Nonuser (no screening in past 2 years), single user (once in the last 2 years), regular user (at least 2 screening mammograms at least 11 months apart in past 2 years)	65–74 v 75 and older	Total 11,039. 75+ = 6813 65–74 = 4226	No follow-up Collection period: 1995–1996	Serious
Richman [50]	USA	Women in the SEER database who had not received a diagnosis of breast cancer before 2002, but had had a screening mammogram in 2002 and were linked with Medicare data. Aged 70 years and older	Cohort study	Population setting, screening mammography and breast cancer diagnosis recorded via population data sets (Medicare and SEER database)	Screening mammograms identified in Medicare claims. Initial screen in 2002, subsequent screen in the 3 year period after 2002 screen	Subsequent screen v no subsequent screen (75–84 years)	23,613 screened, 5707 unscreened	Follow-up: Median 10 years (IQR 5.8–13.9 years) Collection period: 2002–2017	Moderate
Schousboe [39]	USA	65–90 years	Modelling study	Markov modelling using SEER data from 2011 to 2016	Biennial	Stopping screening at 75 compared to 80, 85 and 90 - by CCS, biennial mammography	N/A	N/A	Moderate
Simon [40]	USA	Women’s Health Initiative study—75 years and older	Cohort study	Women in WHI diagnosed with breast cancer	Mammography but no further details	Mammography interval - <2 years, 2–5 years, 5+ years or none	8663 in total - not stated for 75+	Follow-up: average of 12.2 years Collection period: Not stated (WHI study)	Serious
Simon [41]	USA	Women’s Health Imitative study—aged 50–79, post-menopausal, (75 years and older)	Cohort study	Women diagnosed with invasive breast cancer at 75 years and older. Followed up until last document contact, death or September 2010 which ever came first	Clinical trials - Annual or biannual, Observational: at the discretion of the individual	Mammography interval - <2years, 2–5 years, 5+ years or none	Total 1914: <2 years = 1398, 2-5 years = 372 5+ years = 144	Follow-up: mean 4.4 years, max 15.3 years Collection period: Recruitment 1993–1998, Follow-up to 2010	Serious
Smith-Bindman [42]	USA	All women with Medicare	Cohort study	Linked data from Medicare screening services and State cancer registry	Any screening within 1992 and 1993	No screening	Total *N* = 201,537 (75–79 years), screened *n* = 201,537 (40%)	Follow-up: 12-24 months Collection period: 1992–1993	Serious
Upneja [43]	USA	67–74 years	Cohort study	SEER database— women who were screened during 2015	One screening mammogram in 2015	67–74 vs 75 and older (all undergoing 2D mammography)	364,254 vs 230,685	Follow-up: 4 months Collection period: 2015–2016	Serious
VanDijck [44]	The Netherlands	Women invited to screening at least twice, with most recent invitation having occurred over 65 years old	Case control study	Population based screening programme	Women invited to biennial single-view mammography screening. At least 2 mammography screening invitations one of which occurred when the woman is aged 65 years or older	Attended last screen or not	33 cases, 165 referents - from 65 years older - Based on Table 2: 12 cases (and 12*5 = 60 referents)	Follow-up: NA Collection period: cases had died of breast cancer between 1977 and 1988.	Serious
VanDijck [45]	The Netherlands	Nijmegen programme: women invited to screening at least twice, with most recent invitation having occurred over 65 years old	Cohort study	Primary breast cancer patients diagnosed before Dec 1994 aged 50+	Women invited to biennial single-view mammography screening. Women aged 70 years and older invited from 9th screening round onwards	First screen, repeat screen, interval CA or non-participant	4253 invited to first screen and 33,949 invited to subsequent screens	Follow-up: 2 years (for older ages) Collection period: 1975–1994	Serious
VanDijck [46]	The Netherlands	Nijmegen programme: women invited to screening at least twice, with most recent invitation having occurred over 65 years old. Cases died of breast cancer before Jan 1, 1994	Case control study	Population based screening programme,	Women invited to biennial single-view mammography screening. Women aged 70 years and older invited from 9th screening round onwards	No screening (no participation in index round and 4 preceding rounds) Regular screening (participation in index round and negative mammogram in preceding round) Otherwise (not meeting the criteria for unscreened or screened)	Aged 64+: 82 cases, 410 controls aged 75+: 35 cases, 175 controls - no screening 20/97 - regular screening 3/14 - otherwise 12/64	Follow-up: NA Collection period: 1975-1994	Serious
vanRavesteyn [46]	USA	Simulation of a cohort of American women born in 1960, starting screening 50	Modelling study	Simulation	All women received biennial screening starting at 50, with cessation ages varying from 74 up to 96 years	Stopping screening at 74 v continuing screening	N/A as this is statistical modelling	N/A	Low
Vyas [48]	USA	39,006 women aged 70+ with incident BC from 2005 to 2009	Retrospective cohort study	From the Surveillance, Epidemiology and End Results (SEER) Medicare dataset. Programme collects information on newly diagnosed cancer cases from 18 population-based tumour registry’s and covers approx. 26% of the US population.	Mammography screening persistence during the 5 years period before BC diagnosis was to be determined, women who were continuously enroled in Medicare parts A/B for at least 60 months before BC diagnosis, and who were not enroled in health maintenance organisations. Persistent users represented a population who have had annual to biennial mammography screening before BC diagnosis	70–74 v 75–79 v 80 and older	All: 70–74: 12163, 75–79: 11,182, 80+: 15,661. Persistent: 70–74: 6504 75–79: 5672, 80+: 5732 Non-Persistent: 70–74: 3270 75–79: 3019, 80+: 3933 Non-users: 70–74: 2389 75–79: 2491, 80+: 5996	Follow-up: up to 5 years (at least 60 months) Collection period: Incident BC between 2005–2009, and 5 years prior for screening	Serious
Yang [49]	USA	Population data women aged 40 years and older	Cohort study	Pre-1977 historical cohort as America initiated screening in 1977. BC diagnosed in 1999 represents the screening cohort— using SEER data	Mammography according to the ACS guidelines at the time for the age group. Population screening in USA— data from SEER data.	1973–1976 cohort vs 1999 cohort (No screening vs screening)	Total *N*: 10,538 5708 vs 4830	Follow-up: 15 years for survival Collection period: year of diagnosis: 1973–1999	Moderate

^a^To assess risk of bias, we used the Risk of Bias in Non-randomised studies of interventions (ROBINS-I) tool or the Quality of primary diagnostic accuracy studies (QUADAS-2) tool for observational studies and adapted the Risk of Bias tool developed by Carter et al. [15] for modelling studies using two of their criteria (i. transparent assumptions and v. data validation).

### Study registration

This review was prospectively registered with PROSPERO (CRD42020203131).

## Results

**Fig. 1:**
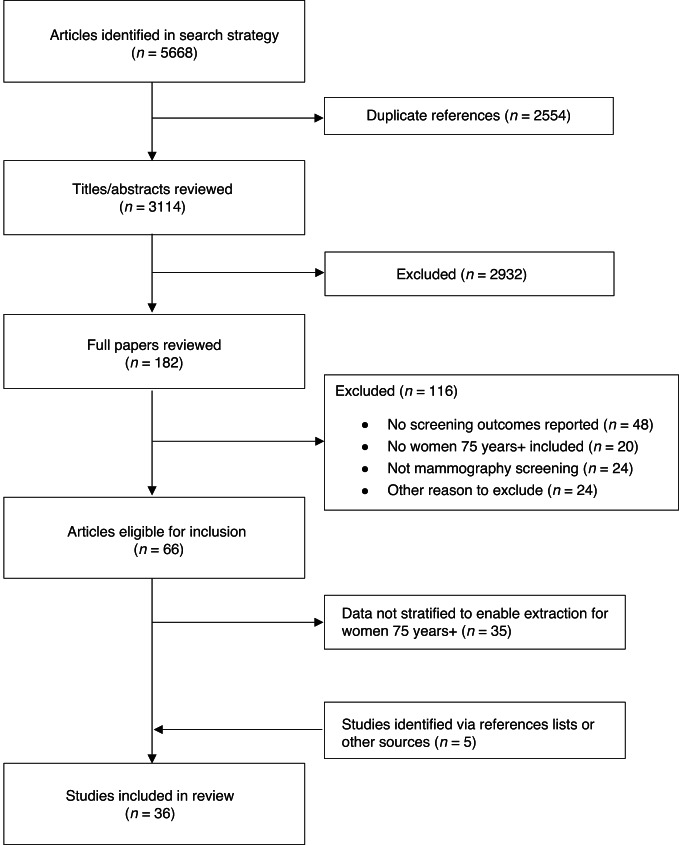
Study identification and selection.

The search strategy yielded 3114 unique titles (Fig. 1). We excluded 2932 records after titles or abstract screening, leaving 182 papers for full-text screen. After full-text screen we excluded 116 publications that did not meet the inclusion criteria. Sixty-six papers were deemed potentially eligible for inclusion. An additional 35 were excluded because data was not stratified for 75 years and older to enable data extraction. Searches of reference lists and additional sources identified 5 additional papers, resulting in a total of 36 studies included in this review [4, 16–50].

Table 1 summarises study characteristics. All included studies contained either observational (*n* = 27) or modelled/simulated data (*n* = 9). No RCTs which evaluated mammography screening in women 75 years and older were identified. As such, all included observational studies were subject to potential selection bias, confounding and lead time bias, with the vast majority of observational studies deemed at critical or serious risk of bias and only two studies at moderate risk of bias. Of the modelling studies, most did not report the assumptions made within the model, and a few failed to describe the validation process (Supplementary Appendix 2). As such, two were deemed low risk of bias, six at moderate risk, and one at high risk of bias.

Of the 27 observational studies, 18 studies followed up women after screening and/or diagnosis; [4, 16, 21, 23, 26, 32, 33, 35–37, 40–43, 45, 48–50] follow-up times in these studies ranged from 4 months to 20 years. Three studies did not include any comparison group and provided only descriptive statistics [22, 24, 27] and three studies compared groups by detection methods in those with breast cancer [32, 33, 36]. One study had multiple comparisons (by age and screening history) [35]; the remaining studies compared mammography screening outcomes in women aged 75 years and older with screening outcomes in women: of other ages (ten studies) [4, 16, 18, 20, 21, 23, 25, 38, 43, 48]; differing screening histories (three studies) [44–46]; differing screening intervals (three studies) [37, 40, 41] or historical unscreened women (one study) [49]; women who did not screen (one study) [42] and women who stopped screening (two studies) [26, 50]. (Table 1).

Of the nine modelling studies included, comparisons and screening histories varied: no comparisons (two studies) [28, 29], comparison of different screening recommendations (one study) [17], comparing with women who have stopped screening (four studies) [30, 34, 39, 47], and comparing screening women of different ages (two studies) [19, 31]. (Table 1).

Outcomes reported in each study varied widely. We have therefore classified outcomes across studies into five groups: measures of health benefits (Table 2); measures of screening harms (Table 3); screening detection measures (Table 4); cancer characteristics (Table 5); and treatment patterns (Table 6). Not all studies presented outcomes in each of these categories.

**Table 2:** Measures of health benefit.

Study Reference	Comparison made	Outcome 1	Data	Outcome 2	Data	Outcome 3	Data
Breast cancer mortality							
Demb [23]	66–74 vs 75–84 vs 85-94	10-year cumulative incidence of breast cancer-related death	0.24% (0.21–0.27%) vs 0.29% (0.25–0.34%) vs 0.31% (0.21–0.43%)	10-year risk of breast cancer death by Charlson Comorbidity index (CCI)	Ages 66–74: CCI0: 0.2% (0.2–0.3%), CCI1: 0.3% (0.2–0.4%), CCI ≥ 2: 0.3% (0.2–0.4%); Ages 75–84: CCI0: 0.3% (0.2–0.3%), CCI1: 0.4% (0.3–0.5%), CCI ≥ 2: 0.3% (0.2–0.5%).		
Garcia-Albaniz [20]	Continue vs stop screening	8-year risk and risk difference for breast cancer death (per 1000 women)	Risk: 3.8 (2.7-5.1) vs 3.7 (3.0-4.6) Risk difference: 0.07 (−0.93 to 1.3)	Breast cancer death hazard ratio	1.00 (0.83–1.19)	8-year risk for breast cancer (%)	5.8% vs 3.9%
Kregting [30]	Annual screening 40-75 to annual screening 40-84	Breast cancer deaths averted	6.9 v 7.2 per 1000 women				
Malmgren [32]	Mammography detected v physician detected v patient detected	Breast Cancer mortality (Stage I-III) age and treatment adjusted	HR = 0.50 (0.31–0.82) *p* < 0.001				
Malmgren [33]	Mammography detected v physician detected v patient detected	Breast cancer mortality (Stage I–III)	HR = 0.38 (0.24–0.61, *p* < 0.001) (mammo decreased mortality - age adjusted)	Breast cancer mortality (Stage I-III)	HR = 2.62 (1.65–4.16, *p* < 0.001) (among pts with PtD and PhyD - age adjusted)		
Mandelblatt [34]	Stopping screening	% Reduction in BC mortality—stopping at 79 vs 69. Median across all models	Annual: 8%, Biennial: 7%	Difference in breast cancer deaths averter per 1000 women— stopping at 79 vs 69. Median across all models	Annual: 2, Biennial: 2		
McCarthy [35]	Non-users compared to regular users	Breast cancer mortality (assuming lead time of 1.25 years for regular users) -	HR: 75–84: 2.47 (1.70– 3.58) 85 +: 1.45 (0.63– 3.32)				
Park [37]	Never/less frequent v biennial v annual screening	Breast cancer specific mortality rates never/less frequent as referent (multivariable analysis)	HR (95%CI) - Biennial: 0.67 (0.31–1.44) Annual: 0.76 (0.38–1.53)				
Richman [50]	Subsequent screen v no subsequent screen	Breast cancer mortality per 100 women (95%CI)	0.36 (0.29–0.46) v 0.42 (0.28-0.64)	Breast cancer mortality HR	0.87 (0.55–1.37)		
Schousboe [39]	Stopping screening at 75 years compared to 80 years, 85 years and 90 years—by CCS, biennial mammography	Breast cancer deaths averted per 1000 women	CCS0: 80y: 1.7 (1.2–2.2) 9.5% 85 y: 2.8 (2.0–3.6) 15.6% 90 y: 3.5 (2.5–4.4) 3.5% CCS1: 80 y: 1.4 (0.9–1.9) 10.4% 85 y: 2.3 (1.5–2.9) 16.9% 90 y: 2.7 (1.9–3.5) 20.3% CCS > = 2; 80 y: 1.0 (0.7–1.4) 11.0% 85 y: 1.7 (1.1–2.1) 17.5% 90 y: 2.0 (1.4–2.5) 20.5%				
Simon [40]	Mammography interval - <2 years, 2–5 years, 5+ years or none	Death from breast cancer (<2 ys reference group) HR	2–5 years mamm HR: 1.87 (1.10–3.19) 5+ or no mamm HR: 3.17 (1.68-5.96) p-trend = 0.0001				
Simon [41]	Mammography interval - <2 years, 2–5 years, 5+ years or none	Death from Breast cancer (<2 ys reference group)	2-5years HR = 1.62 (1.03–2.54) 5+ years or none HR: 2.80 (1.57–5.00) ptrend = 0.0002	Subdistribution HR for breast cancer death taking competing causes into account	SHR for 2–5 years: 1.60 (1.02–2.52), 5+years or none: 2.74 (1.50–5.03)		
Van Dijck [44]	Attended last screen or not	Breast cancer mortality rate ratio aged 75+, cases attended yes v no	RR: 2.87 (95%CI 0.62-13.2) (increased risk in those who attended screening)	Observed/expected breast cancer death ratio in a town with screening programme compared to a town without.	75–79: 0.92, 80+: 0.29		
Van Dijck [46]	No screening v Regular screening v Otherwise	Ratio of breast cancer mortality rates of the women who had participated regularly (2 most recent screening rounds prior to diagnosis) vs women who had not participated	Rate ratio: 1.05 (0.27–4.14)	Ratio of breast cancer mortality rates of the ‘otherwise’ women vs women who had not participated	0.90 (0.40–2.02)		
All Cause/Other Cause Mortality							
Arleo [17]	Different screening recommendations	Deaths averted per 1000 women Mean (Median)	A75–79:1.05 (1.35) A75–84: 1.9 (2.3) B75-79: 0.9 (1.05) B75–84: 2.0 (2.0)	% Mortality reduction: mean (median)	A75-79: 3.5 (4.5) A75-84: 6.3 (7.5) B75-79: 3.0 (3.5) B75-84: 5.3 (6.5)		
Demb [23]	66–74 vs 75–84 vs 85–94	10-year cumulative incidence of non-breast cancer-related death	14.5% (14.3–14.8%) vs 35.7% (35.3–36.1%) vs 65.4% (64.3–66.5%)	Among women who were not diagnosed with breast cancer, 10-year risk of non-breast cancer-related death by Charlson Comorbidity index (CCI)	Ages 66–74: CCI0: 10.4% (10.3–10.7%), CCI1: 22.5% (21.9–23.1%), CCI ≥ 2: 43.4% (42.2–44.4%); Ages 75–84: CCI0: 29.8% (29.3–30.2%), CCI1: 46.0% (45.1–47.0%), CCI ≥ 2: 61.7% (60.2–63.3%); Ages 85–94: CCI0: 60.3% (59.1–61.5%), CCI1: 72.8% (70.6–74.7%), CCI ≥ 2: 84.8% (82.5–86.9%).		
McPherson [36]	Mammography detected v clinically detected cancers	RR of death (comparison is clinically detected cancers)	75–79: 0.38 (0.25–0.44); 80–84: 0.75 (0.57–0.99); 85+: 0.67 (0.50–0.90)				
Richman [50]	Subsequent screen v no subsequent screen	% of cohort who died during follow up period (75-84 year olds at baseline)	65% v 80%				
Schousboe [39]	Stopping screening at 75 compared to 80, 85 and 90 years old - by CCS, biennial mammography	Years of life gain - biennial mammography	CCS0: 80 y: 16.0 (11.7–21.0) 85 y: 24.7 (18.4–31.3) 90 y: 28.3 (21.6–35.5) CCS1: 80 y: 11.5 (9.6–14.9) 85 y: 17.3 (13.6–22.6) 90 y: 19.4 (15.0–25.7) CCS > = 2; 80 y: 7.5 (5.1–10.1) 85 y: 10.8 (7.5–15.3) 90 y: 12.1 (8.2–17.2)				
Simon [41]	Mammography interval <2 years (reference group for all comparisons), 2–5 years, 5+ years or none	Death from other causes	2–5 years HR: 1.08 (0.77–1.50), 5+ years or none HR: 1.33 (0.83–2.15) p-trend = 0.25	Death from any cause	2–5 years HR: 1.23 (0.94–1.61) 5+ years or none: HR 1.73 (1.20–2.50) p-trend=0.002	Subdistribution HR for all cause death taking competing causes into account	SHR for 2–5 years: 1.01 (0.71–1.44), 5+ years or none: 1.22 (0.78–1.92) p-trend=0.49
Life Years Gained							
Arleo [17]	Different screening recommendations	Life years gained per 1000w mean(median)	A75–79: 9 (7) A75–84: 14 (11) B75–79: 7 (7) B75–84: 11 (12)	NNS per life yr. gained mean (median)	A75–79: 111 (143) A75–84: 71 (91) B75–79: 143 (143) B75–84: 91 (83)	LYG/death averted mean	A75–79: 8.6 A75–84: 7.4 B75–79: 7.8 B75–84: 6.5
Boer [19]	Optimistic variant vs pessimistic variant	Life-years gained (* 1000)	upper age limit at 69: 408 vs 395; upper age limit at 79: 480 vs 476; upper age limit at 81: 486 vs 483; upper age limit at 89: 494 vs 496; upper age limit at 99: 497 vs 497.	Life-years in lead time (* 1000)	upper age limit at 69: 357 vs 377; upper age limit at 79: 517 vs 703; upper age limit at 81: 538 vs 776; upper age limit at 99: 597 vs 1057.		
Boer [19]	69 vs 99	Life-years gained (* 1000)	292 vs 292	Life-years in lead time (* 1000)	275 vs 463		
Jansen [28]	No comparison	Total lifetime gained (by annual screening one million women)	65–69: 5196 years; 70–74: 3088 years; 75–79: 1489 years; 80–84: 546 years; 85–89: 134 years; 90–94: 15 years; 95–99: 0 years.				
Lansdorp-Vogelaar [31]	74 vs 76	Life-year gained (per 1000 screen) MISCAN-Fadia model	Average health: 7.6 vs 6.9; No comorbidity: 8.5 vs 7.8; Mild comorbidity: 6.9 vs 6.1; Moderate comorbidity: 6.2 vs 5.3; Severe comorbidity: 4.5 vs 4.0;	Life-year gained (per 1000 screen) G-E model	Average health: 5.8 vs 5.1; No comorbidity: 6.6 vs 5.6; Mild comorbidity: 5.1 vs 4.2; Moderate comorbidity: 4.8 vs 3.8; Severe comorbidity: 3.5 vs 2.9;		
Mandelblatt [34]	Stopping screening	Difference in life years gained per 1000 women - stopping at 79 vs 69. Median across all models	Annual: 24, Biennial 23.5				
vanRavesteyn [46]	Stopping screening at 74 compared to continuing screening	Life years gained	7.8–11.4 LYG per 1000screens (screening a woman at age 74) 4.8–7.8LYG per 1000 screens (Screening a woman at age 80) 1.4–2.4 LYG per 1000 screens (Screening a woman at age 90)	Breast cancer death averted and LYG	A breast cancer death averted at: age 74 saves 15.4-17.9 life years age 80: 6.5–7.7 life years age 90: 3.0–3.7 life years		
Survival							
Malmgren [32]	Mammography detected v physician detected v patient detected	5-year disease specific survival	98% MgD v 87% % PtD (*p* < 0.001)	10-year disease specific survival	92% MgD v 80% PtD (*p* < 0.001)		
McCarthy [35]	Age at diagnosis	5-year survival by stage -	Stage 1: 67–74: 0.877 (0.013) 75-84: 0.842 (0.016) 85+: 0.496 (0.052) *p* = 0.0001 Stage 2: 67–74: 0.785 (0.021) 75-84: 0.620 (0.026) 85+: 0.345 (0.043) *p* = 0.001 Stage 3/4 67–74: 0.362 (0.040) 75–84: 0.286 (0.036) 85+: 0.225 (0.055) *p* = 0.039				
McPherson [36]	Mammography detected v clinically detected cancers	Overall survival	MgD: 75–79 0.69, 80-84 0.45, 85 + 0.46; ClinD: 75–79 0.33, 80-84 0.39, 85 + 0.17.	Relative survival (MgD v ClinD)	75–79: 1.00 v 0.54; 80–84: 0.89 v 0.76; 85+: 1.00 v 0.39		
Yang [49]	1973–1976 cohort vs 1999 cohort (No screening vs screening)	15-year cumulative survival rates between early-stage breast cancer cohorts	Cumulative survival rate: 10.9% v 18.6% - Improvement in survival rate: 7.7%				
Other Mortality measures							
Jansen [29]	No comparison	Net effect screening (reduction in the number of fatal breast tumours due to screening, taking into account the risk of inducing a fatal tumour)	75–79: 269 80–84: 141 85–89: 54 90–94: 12 95–99: 1				
Lansdorp-Vogelaar [31]	74 vs 76 - MISCAN-Fadia model	Cancer deaths prevented (per 1000 screen)	Average health: 0.9 vs 0.9; No comorbidity: 1.0 vs 1.0; Mild comorbidity: 0.8 vs 0.9; Moderate comorbidity: 0.8 vs 0.8; Severe comorbidity: 0.6 vs 0.6;	The number needed to screen to gain one life-year	Average health: 132 vs 146; No comorbidity: 117 vs 128; Mild comorbidity: 146 vs 163; Moderate comorbidity: 162 vs 189; Severe comorbidity: 223 vs 253;	The number needed to screen to prevent one cancer death	Average health: 1125 vs 1102; No comorbidity: 1039 vs 1007; Mild comorbidity: 1192 vs 1165; Moderate comorbidity: 1251 vs 1263; Severe comorbidity: 1612 vs 1612;
Lansdorp-Vogelaar [31]	74 vs 76 - G-E Model	Cancer deaths prevented (per 1000 screen)	Average health: 0.7 vs 0.7; No comorbidity: 0.8 vs 0.7; Mild comorbidity: 0.7 vs 0.6; Moderate comorbidity: 0.6 vs 0.6; Severe comorbidity: 0.5 vs 0.5;	The number needed to screen to gain one life-year	Average health: 173 vs 198; No comorbidity: 150 vs 179; Mild comorbidity: 197 vs 237; Moderate comorbidity: 209 vs 266; Severe comorbidity: 286 vs 348;	The number needed to screen to prevent one cancer death	Average health: 1421 vs 1474; No comorbidity: 1269 vs 1368; Mild comorbidity: 1514 vs 1612; Moderate comorbidity: 1712 vs 1947; Severe comorbidity: 2102 vs 2207;
QALY/Cost Effectiveness ratio							
Boer [19]	Optimistic variant vs pessimistic variant	Cost-effectiveness ratio	69–75 years: £8400 per QALY gained; 69–79 years: £36,000 per QALY gained				
Boer [19]	69 vs 99	QALYs gained (5% discounted)	62,727 vs 57,855				
Kregting [30]	Annual screening 40–75 v annual screening 40-84	QALYs gained	96.1 v 97.7 per 1000 women				
Schousboe [39]	Stopping screening at 75 compared to 80, 85 and 90 - by CCS, biennial mammography	QALYS gained - Biennial mammography	CCS0: 80 y: 10.9 (7.1–14.4) 85 y: 16.0 (10.8–21.0) 90 y: 17.6 (11.8–23.2) CCS1: 80 y: 7.3 (4.5–10.6) 85 y: 10.3 (6.6–15.1) 90 y: 11.3 (6.8–16.7) CCS > = 2; 80 y: 4.9 (2.9–6.8) 85 y: 6.8 (4.0–9.5) 90 y: 7.2 (4.1–10.3)				
vanRavesteyn [46]	Stopping screening at 74 compared to continuing screening	QALYS gain per 1000 screens	at age 74: 7.1–9.9 at age 80: 4.0–6.1 At age 84: 2.4–3.7

**Table 3:** Screening harms.

Study Reference	Comparison made	Outcome 1	Data	Outcome 2	Data
False positives and recalls					
Arleo [17]	Annual and biennial 75–79 and 75–84	Recalls per 1000 women screened	A75–79: 170 A75–84: 310 B75–79:80 B75–84:190		
Bennett [18]	70–74 v 75 and older	Recall for assessment	4177 (4.8%) vs 2968 (5.6%)		
Braithwaite [21]	66–74 vs 75–89	Woman-level cumulative false-positive recall after 10 years of repeat screening	Charlson Score=0 & first mammography: 8.6% (8.3–8.8%) vs 8.0% (7.6–8.4%); Charlson score = 0 and 1-year screen interval: 49.7% (47.8–51.5%) vs 47.2% (44.9–49.5%); Charlson Score=0 & 2-year screen interval: 30.2% (29.4–31.1%) vs 26.6% (25.7–27.5%); Charlson score ≥ 1 & first mammography: 8.9% (8.5–9.3%) vs 8.8% (8.2–9.4%); Charlson score ≥ 1 & 1-year screen interval: 48.0% (46.1–49.9%) vs 48.4% (46.1–50.8%); Charlson score ≥ 1 & 2-year screen interval: 29.0% (28.1–29.9%) vs 27.4% (26.5–28.4%).	Woman-level cumulative false-positive biopsy recommendation after 10 years of repeat screening	Charlson Score=0 & first mammography: 1.2% (1.1–1.3%) vs 1.2% (1.1–1.4%); Charlson Score=0 & 1-year screen interval: 9.8% (8.4%-11.3%) vs 9.2% (7.5–11.2%); Charlson Score=0 & 2-year screen interval: 4.6% (4.2–5.1%) vs 4.1% (3.7–4.6%); Charlson score≥1 & first mammography: 1.7% (1.5%-1.9%) vs 1.7% (1.4–2.0%); Charlson score≥1 & 1-year screen interval: 11.8% (10.1–13.8%) vs 11.3% (9.3–13.6%) Charlson score≥1 & 2-year screen interval: 5.6% (5.1–6.2%) vs 5.1% (4.5–5.7%).
Kregting [30]	40–75 v 40–84 (annual screening)	False positives	216 v 229 per 1000 women		
Lansdorp-Vogelaar [31]	74 vs 76	False-positive tests (per 1000 screen) MISCAN-Fadia model	Average health: 79 vs 77 No comorbidity: 79 vs 77 Mild comorbidity: 79 vs 77 Moderate comorbidity: 79 vs 77 Severe comorbidity: 79 vs 77	False-positive tests (per 1000 screen) G-E Model	Average health: 96 vs 96; No comorbidity: 95 vs 95; Mild comorbidity: 95 vs 95; Moderate comorbidity: 95 vs 95; Severe comorbidity: 97 vs 98;
Schousboe [39]	Stopping screening at 75 compared to 80, 85 and 90—by CCS, biennial mammography	False positive screen results	CCS0: 80 y: 165 (133–192) 85 y: 301 (243–351) 90 y: 389 (314–454) CCS1: 80 y: 147 (118–175) 85 y: 259 (208–308) 90 y: 325 (262–387) CCS > = 2; 80 y:107 (89–127) 85 y: 182 (151–216) 90 y: 255 (188–268)	Additional screens - biennial mammography	CCS0: 80 y: 2007 (1977–2035) 85 y: 3672 (3608–3733) 90 y: 4885 (4790–4977) CCS1: 80 y: 1739 (1716–1765) 85 y: 3064 (3013–3118) 90 y: 3929 (3853–4006) CCS > = 2; 80 y: 1371 (1352–1391) 85 y: 2333 (2293–2374) 90 y: 2989 (2880–2953)
Upneja [43]	67–74 vs 75+	Subsequent imaging (adjusted)	100.3(96.1–104.5) vs 97.0(91.8–102)		
Biopsy					
Advani [16]	66–74 vs 75–84 vs 85–94	Rate of screening mammogram followed by biopsy (per 1000 screens)— any biopsy	15.7 (14.8–16.8) vs 14.5 (13.5–15.6) vs 13.2 (11.3–15.4) p-trend <0.001	Prevalence of any biopsy	1.6% vs 1.5% vs 1.4%
Arleo [17]	Annual and biennial 75–79 and 75–84	Benign biopsies per 1000 women screened	A75–79: 12 A75–84: 22 B75–79: 5 B75–84: 13		
Braithwaite [20]	66–74 vs 75–84 vs 85–99	Biopsy rate after screening	1.3% vs 1.2% vs 1.2% (p-trend = 0.07)	Proportion of screens followed by biopsy by Charlson Comorbidity score (CCS)	Ages 66–74: CCS0:0.77%, CCS1:0.88%, CCS ≥ 2:0.94% (p-trend<0.001); Ages 75–84: CCS0:0.62%, CCS1:0.75%, CCS ≥ 2:0.78% (p-trend=0.001); Ages 85–99: CCS0:0.48%, CCS1:0.57% and CCS ≥ 2:0.61% (p-trend = 0.23).
Hartman [27]	75 years and older (no comparison)	Biopsy rate	1.4% (60/4424)		
Schousboe [39]	Stopping screening at 75 compared to 80, 85, and 90—by CCS, biennial mammography	False positive biopsy results	CCS0: 80 y: 14.7 (12.0–17.2) 85 y: 26.9 (21.9–31.5) 90 y: 33.7 (27.5–39.4) CCS1: 80 y: 14.3 (11.6–17.0) 85 y: 25.2 (20.4–29.9) 90 y: 30.6 (24.8–36.4) CCS > = 2; 80 y:12.9 (10.7–15.4) 85 y: 22.0 (18.2–26.2) 90 y: 26.4 (21.8–31.5)		
Upneja [43]	67–74 vs 75+	Biopsy rate(adjusted)	16.1(14.7–17.6) vs 16.8(15.0–18.5) per 1000 mammograms		
Overdiagnosis					
Boer [19]	Optimistic variant vs pessimistic variant	Extra incidence (cases)	Upper age limit at 69: 5266 vs 6258; Upper age limit at 79: 12205 vs 25142; Upper age limit at 81: 13864 vs 32356; Upper age limit at 99: 22288 vs 77760.		
Boer [19]	69 vs 99	Extra incidence (cases)	4651 vs 17663		
Kregting [30]	Annual screening 40–75 v Annual screening 40–84	Overdiagnosis	7.3 v 8.5 per 1000 women		
Lansdorp-Vogelaar [31]	74 vs 76	Over-diagnosed cancers (per 1000 screen) MISCAN-Fadia model	Average health: 0.8 vs 1.0; No comorbidity: 0.5 vs 0.7; Mild comorbidity: 0.8 vs 1.1; Moderate comorbidity: 0.9 vs 1.3; Severe comorbidity: 1.9 vs 2.2;	Over-diagnosed cancers (per 1000 screen) G-E Model	Average health: 0.5 vs 0.6; No comorbidity: 0.3 vs 0.4; Mild comorbidity: 0.5 vs 0.7; Moderate comorbidity: 0.6 vs 0.8; Severe comorbidity: 1.3 vs 1.5;
Richman [50]	Subsequent screen v no subsequent screen	% of breast cancer cases among screened women aged 75–84 years overdiagnosed	47%		
Schousboe [39]	Stopping screening at 75 compared to 80, 85 and 90 - by CCS, biennial mammography	Number of overdiagnosed cases per 1000 women screened	CCS0: 80 y: 3.2 (1.8–4.7) 85 y: 5.4 (3.2–8.0) 90 y: 7.0 (4.1–10.2) CCS1: 80 y: 2.8 (1.2–3.9) 85 y: 4.6 (2.2–6.5) 90 y: 5.7 (2.8–8.1) CCS > = 2; 80 y:2.2 (1.3–3.2) 85 y: 3.6 (2.1–5.1) 90 y: 4.3 (2.5–6.2)		
vanRavesteyn [46]	Stopping screening at 74 compared to continuing screening	Over diagnosed breast cancers	1.2–5 .0 per 1000 screens at age 74, compared to 1.8 to 6.0 at age 80 years, and 3.7 to 7.5 at age 90 years.	Overdiagnosis as a %	5–32% (screening 50–74), 14–36% (for a screen at 80) and 28–41% for a screen at 90
Yang [49]	1973–1976 cohort vs 1999 cohort (No screening vs screening)	Excess early-stage cancer	54.1% v 76.4–22.4% excess *p* < 0.001	The proportion of age specific overdiagnosis (75 years and older)	8.3% of breast cancers in women aged 75 yrs and older were over diagnosed.

**Table 4:** Screening detection measures^a^.

Study Reference	Comparison Made	Outcome 1	Data	Outcome 2	Data
Cancer detection rates					
Cate [22]	No comparison	Cancer detection rate	4.9 per 1000 screening examinations (10 of 2057 patients)		
Destounis [24]	No comparison	Cancer detection	8.4 per 1000 exams		
Hartman [27]	No comparison	Cancer detection rate	5.9 per 1000 screens		
Richman [50]	Subsequent screen v no subsequent screen	Cumulative incidence of breast cancer per 100 women (95%CI)	4.85 (4.57–5.15) v 2.56 (2.20–2.97)	Risk difference	2.29 (1.74–2.81)
Smith-Bindman [42]	No screening	RR of breast cancer	3.6 (3.3–4.0)		
Upneja [43]	67–74 vs 75+	Total cancer diagnosis	7.3(6.9–7.6) vs 9.4(8.8–9.9) per 1000 women screened		
Invasive detection rates					
Bennett [18]	70–74 v 75 and older	Invasive cancers	1073 (12.4/1000) vs 815 (15.3/1000)	Invasive cancers <15 mm	A: 537 (6.2/1000) B: 395 (7.4/1000)
Braithwaite [21]	66–74 vs 75–89	Invasive cancers	Charlson Score=0 & 1-year screen interval: 81.6% vs 81.1%; Charlson Score=0 & 2-year screen interval: 80.1% vs 84.8%; Charlson Score=1 & 1-year screen interval: 77.8% vs 86.0%; Charlson Score=1 & 2-year screen interval: 76.9% vs 86.4%.	Invasive cancer number	All cancers: 1346 vs 1096. Charlson Score=0 & 1-year screen interval: 672 vs 490; Charlson Score=0 & 2-year screen interval: 323 vs 308; Charlson Score=1 & 1-year screen interval: 231 vs 190; Charlson Score=1 & 2-year screen interval: 120 vs 108.
Demb [23]	66–74 vs 75–84 vs 85–94	10-year cumulative incidence of invasive breast cancer	4.0% (3.9–4.1%) vs 3.6% (3.5–3.8%) vs 2.7% (2.4–3.0%)	10-year risk of invasive breast cancer by Charlson Comorbidity index (CCI)	Ages 66–74: CCI0: 4.0% (3.9–4.2%), CCI1: 4.0% (3.7–4.2%), CCI ≥ 2: 3.9% (3.5–4.3%); Ages 75–84: CCI0: 3.7% (3.5–3.9%), CCI1: 3.4% (3.1–3.7%), CCI ≥ 2: 3.4% (2.9–3.9%); Ages 85–94: CCI0: 2.7% (2.3–3.1%), CCI1: 2.9% (2.2–3.7%), CCI ≥ 2: 2.1% (1.3–3.0%).
Destounis [24]	No comparison	Invasive cancers	82% of all malignancy. 63% were grade 2 or 3.		
El-Zaemey [4]	65–69 v 70–74 v 75 and older	Invasive screen-detected cancer (per 1000 screens; 95% CI)	6.5 (5.8–7.4) v 8.1 (7.1–9.3) v 11.4 (9.3-13.9)		
Erbas [25]	40–49 vs 50–69 vs 70–74 vs 75 and older	Invasive breast cancer rate (per 1000 women screened)	2.49 vs 4.07 vs 5.46 vs 5.14		
Richman [50]	Subsequent screen v no subsequent screen	Localised invasive breast cancer incidence per 100 women (95%CI)	3.15 (2.95–3.38) v 1.50 (1.21–1.86)	Risk difference	1.65 (1.21–2.03)
Smith-Bindman [42]	No screening	RR of Breast cancer	Local:4.4 (3.9–5.0) Regional:3.1 (2.5–3.7) Metastatic: 0.69(0.46–1.0)		
DCIS rate					
Bennett [18]	70–74 v 75 and older	In Situ cancer	227 (2.6 per 1000) vs 139 (2.6 per 1000)		
Braithwaite [21]	66–74 vs 75–89	DCIS	Charlson Score=0 & 1-year screen interval: 18.4% vs 18.9%; Charlson Score=0 & 2-year screen interval: 19.9% v 15.2% Charlson Score=1 & 1-year screen interval: 22.2% v 14.0% Charlson Score=1 & 2-year screen interval: 23.1% v 13.6%		
Demb [23]	66-74 vs 75-84 vs 85-94	10-year cumulative incidence of DCIS	1.0% (0.9–1.0%) vs 0.7% (0.6–0.8%) vs 0.4% (0.3–0.5%)		
El-Zaemey [4]	65-69 v 70–74 v 75 and older	In situ screen-detected cancer (per 1000 screens; 95% CI)	1.6 (1.3–2.1) v 2.0 (1.5-2.7) v 1.3 (0.7-2.2)		
Erbas [25]	40–49 vs 50–69 vs 70–74 vs 75 and older	DCIS rate (per 1000 women screened)	0.83 vs 0.82 vs 0.87 vs 0.97		
Richman [50]	Subsequent screen v no subsequent screen	In situ breast cancer incidence per 100 women (95%CI)	0.79 (0.68–0.93) – 0.15 (0.07–0.29)	Risk difference	0.64 (0.46–0.79)
Smith-Bindman [42]	No screening	RR of DCIS	4.9 (3.5-6.9)		
Interval cancer rates					
El-Zaemey [4]	65–69 v 70–74 v 75 and older	Invasive interval cancer (per 1000 screens; 95% CI)	1.7 (1.3–2.2) v 2.2 (1.8–2.9) v 1.5 (0.9-2.5)		
PPV					
Bennett [18]	70–74 v 75 and older	PPV	31.2%, vs 32.3%		
Garcia-Albeniz [26]	Continue vs stop screening	PPV	41.5% vs 48.4%		
Hartman [27]	No comparison	PPV2	40.6% (26/64)	PPV3	43.3% (26/60)

^a^Cancer detection measures except where specified as cancer rates or cumulative incidence rates

**Table 5:** Cancer characteristics.

Study Reference	Comparison made	Outcome 1	Data	Outcome 2	Data2	Outcome 3	Data3
Node positive							
Braithwaite [21]	66–74 vs 75–89	Positive lymph nodes	Charlson Score=0 & 1-year screen interval: 21.2% vs 16.3%; Charlson Score=0 & 2-year screen interval: 20.6% vs 15.7%; Charlson Score=1 & 1-year screen interval: 24.1% vs 16.6%; Charlson Score=1 & 2-year screen interval: 17.8% vs 12.4%.				
Destounis [24]	No comparison	Positive lymph nodes	7%				
Erbas [25]	40–49 vs 50–69 vs 70–74 vs ≥75	Invasive cancer node positive (%)	28.99% vs 19.51% vs 15.27% vs 9.45% (*p* < 0.001)	Interval cancer node positive (%)	75.40% vs 84.26% vs 71.21% vs 59.26% (*p* < 0.001)		
Malmgren [33]	Mammography v physician v patient detected	Lymph nodes positive	MgD 12% vs PtD+PhysD 38%				
Stage Distribution							
Braithwaite [21]	66–74 vs 75–89	Advanced stage (IIB-IV)	Charlson Score=0 & 1-year screen interval: 12.0% vs 9.4%; Charlson Score=0 & 2-year screen interval: 10.9% vs 10.7%; Charlson Score=1 & 1-year screen interval: 15.8% vs 11.7%; Charlson Score=1 & 2-year screen interval: 11.1% vs 5.7%.				
Erbas [25]	40–49 vs 50–69 vs 70–74 vs 75 and older	Invasive cancer grade III (%)	20.29% vs 18.10% vs 15.06% vs 13.39% (*p* = 0.05)	Interval cancer grade III (%)	49.06% vs 38.18% vs 30.91% vs 45.45% (*p* = 0.06)		
Hartman [27]	No comparison	Stage	Stage was known for 17 of 26 women: 88% (15/17) had stage 0 (n = 3) or stage I (*n* = 12) disease and 12% (*n* = 2) had stage II disease. Stage 3: 0. Unknown stage: 9 (35%)	Grade	Unknown: 1; High: 5; Intermediate to high: 3; Intermediate: 11; Low to intermediate: 3, Low: 3.		
McCarthy [35]	Non users v regular users	Late-stage disease	75–84 (*n* = 1790) OR 3.64 (2.96-4.48) 85+ (*n* = 455) OR 6.87 (3.97–11.90) Adjusted for age at diagnosis, race, marital status, income of ZIP code and comorbidity				
Vyas [48]	70–74 v 45–79 v 80 and older	Diagnosis at insitu stage	70–74: 1 (reference group) 75–79: 0.97 (0.84–1.11) 80+: 0.79 (0.69–0.90)	Diagnosis at local stage	70–74: 1(reference group) 75–79: 1.02 (0.90–1.16) 80+: 1.13 (1.00–1.26)	Diagnosis at regional stage	70–74: 1(reference group) 75–79: 1.03 (0.90–1.17) 80+: 1.00 (1.88–1.12)
Tumour size							
Erbas [25]	40–49 vs 50–69 vs 70–74 vs 75 and older	Invasive cancer mean size (mm)	17.2 vs 13.88 vs 13.40 vs 13.66 (*p* = 0.08)	Invasive cancer mean size (mm) by time since previous negative screen	<27 months: 16.97 vs 13.66 vs 13.35 vs 12.71; 27–36 months: 17.25 vs 14.17 vs 13.08 vs 12.41; ≥37 months: 17.51 vs 16.91 vs 15.21 vs 17.46	Interval cancer mean size (mm)	20.64 vs 21.39 vs 18.61 vs 20.75
Malmgren [33]	Mammography detected v physician detected/ patient detected	Mean tumour size (cm)	3.02 (pt/phys) v 1.53 (mammo) *p* < 0.001				
Randolph [38]	65–74 v 75 and older	Mean tumour size (adjusted 69–74 v 75 + - non, single, regular user)	25.5, 19.3, 17.4 v 28.9, 20.6, 16.9.				
VanDijck [45]	First screen, repeat screen, interval CA or non-participant	Tumour size of invasive cancers according to detection round First screening: Repeat screen: Interval: non-participants	Median (25-75 centile); 20(14–27): 12 (7–20): 20 (13–25): 30(20–40)

**Table 6:** Treatment patterns.

Study Reference	Comparison made	Outcome 1	Data	Outcome 2	Data2	Outcome 3	Data3	Outcome 4	Data4	Outcome 5	Data
Destounis [24]	No comparison	Surgically treated	98%								
Garcia-Albeniz [26]	Continue vs stop screening	Lumpectomy	48.8% (47.9%–49.5%) vs 32.6% (31.5%–33.8%)	Radical mastectomy	14.2% (13.7%–14.6%) vs 17.0% (16.0%–17.9%)	Radiotherapy	41.2% (40.4%–41.9%) vs 31.9% (30.7%–33.1%)	Chemotherapy	8.6% (8.3%–9.1%) vs 11.5% (10.6%–12.3%)	simple mastectomy	10.8 (10.3-11.2) v 10.1 (9.4-10.9)
Hartman [27]	No comparison	Treatment (women)	Unknown: 5; lumpectomy: 18 (86%); mastectomy: 1 (5%); hormonal therapy only: 2 (10%).								

### Measures of health benefit

The health benefits of screening in women over 75 years were reported using heterogeneous outcome measures that included breast cancer mortality, all cause/other cause mortality, survival, life years gained, and measures of quality-adjusted life years and cost effectiveness (Table 2).

Of the 14 studies that reported breast cancer mortality, two studies showed a significant decrease in breast cancer mortality among women who had screen-detected breast cancer compared to women who had their cancer detected by other means including those detected clinically; (HR: 0.50 (0.31–0.82) *p* < 0.001, and HR: 0.38 (0.24–0.61), *p* < 0.001) [32, 33]; Simon (2013 and 2014) [40, 41] also demonstrated an increased hazard ratio (HR) as the mammography screen interval increased in women who screen (2–5 years screen interval HR: 1.87 (1.10–3.19), 5+ years screen interval or no mammography HR: 3.17 (1.68–5.96); and 2–5 years screen interval HR: 1.62 (1.03–2.54), 5+ years screen interval or none HR: 2.80 (1.57–5.00). McCarthy [35] showed an increased HR in non-users compared to regular users of mammography screening (HR 75–84 yrs: 2.47 (1.70–3.58)). Schousboe [39] demonstrated a decrease in the number of breast cancer deaths by continuing screening beyond the age of 75, with various estimates stratified by Charlson Comorbidity score (CCS) and age for continuing screening (CCS0: Compared to stopping at 75 yrs, deaths averted per 1000 screens, 80 y: 1.7 (1.2–2.2); 85 y: 2.8 (2.0–3.6); 90 y: 3.5 (2.5–4.4)) .

In contrast, four studies [23, 37, 44, 45] showed no significant differences in breast cancer mortality between screen-detected cancers in women aged 75 years and older and their comparator, comprising younger women (66–74 vs 75–84 years: 0.24% (0.21–0.27%) vs 0.29% (0.25–0.34%)) [23], those who did not attend their last screen (RR: 2.87 (95%CI 0.62–13.2)) [44], those who had not participated (Rate ratio: 1.05 (0.27–4.14)) [45], and those who had never/less frequently attended screening (HR: 0.67 (0.31–1.44)) [37]. One study showed similar estimates for breast cancer deaths averted for those screened annually from 40 to 75 years vs those screened annually from 40 to 85 years (6.9 v 7.2 per 1000 women) [30].

Only two cohort studies compared the risk of breast cancer death between women who stopped screening at 75 years and those who continued [26, 50], both of which reported a non-significant difference in breast cancer mortality. Garcia-Albeniz (2020) reported the 8-year risk of breast cancer death of 0.07 per 1000 women [26] and Richman reported a breast cancer mortality hazard ratio of 0.87 (95% CI 0.55–1.37) [50]. Two microsimulation modelling studies [34, 47] however indicated benefits (in terms of life-years gained (LYG)) for continuing screening into older age (difference in LYG per 1000 women—stopping at 79 vs 69: 23.5 [34], 4.8–7.8LYG per 1000 screens (screening a woman at age 80) [47], as well as a reduction in breast cancer mortality (7% reduction in BC mortality) [34].

All four studies that reported survival showed that screening increased survival (Table 2) [32, 35, 36, 49].

Demb et al. [23] showed that across age-groups (66–74 vs 75–84 vs 85–94) of women who had at least one screen, breast cancer mortality modestly increased (0.24% (0.21–0.27%) vs 0.29% (0.25–0.34%) vs 0.31% (0.21–0.43%), whereas mortality from other causes substantially increased (14.5% (14.3–14.8%) vs 35.7% (35.3–36.1%) vs 65.4% (64.3–66.5%)). Additional outcomes data, including by co-morbidity where reported, are shown in Table 2.

### Measures of screening harms

Measures that were considered screening harms included rates of false positives and recalls, biopsy, and overdiagnosis (Table 3).

In the seven studies that reported on false positives and recalls [17, 18, 21, 30, 31, 39, 43], the proportions of false positive tests and recalls for women aged 75 years and over who attended screening and the comparator group were generally similar, with the exception of two studies showing a significant additional number of false positive screens [39] and false-positive (benign) biopsies [17] associated with continuing screening beyond 75, compared to stopping at the age of 75.

Overdiagnosis was estimated in seven studies (two of which provided multiple comparisons) [19, 30, 31, 39, 47, 49, 50], all of which reported an increase in overdiagnosis from screening older age-groups. However, the estimated magnitude (and defined measure) of increase in overdiagnosed cancers varied substantially across studies, ranging from 0.5 to 0.6 per 1000 screens for women aged 76 years in average health (G-E model) [31] to 47% of breast cancer cases among screened women aged 75–84 years old being overdiagnosed [50].

### Screening detection measures

Screening detection measures included cancer detection rates, invasive cancer detection rates, DCIS rates, interval cancer rates and positive predictive value (PPV) (Table 4); where reported, we also considered cancer incidence rates (Table 4).

Of the six studies that reported cancer detection or diagnosis rates [22, 24, 27, 42, 43, 50], these rates ranged from 4.85 to 9.4 per 1000 screens in women aged 75+. Only three studies provided a comparator group; Upneja [43] reported a total breast cancer diagnosis rate of 9.4 per 1000 for women 75 and older compared to 7.3 per 1000 women aged 67–74; Smith-Bindman [42] compared screening to no screening and provided a relative risk (RR) of breast cancer of 3.6 (95% CI: 3.3–4.0); and Richman [50] compared women aged 75–84 years who continue screening to women who do not, and reported a cumulative incidence of breast cancer of 4.85 per 100 in those who attend screening compared to 2.56 per 100 in those who do not attend further screens.

Five of the eight studies that reported on the detection rate of invasive cancers provided an age-group comparison. The rates were similar or slightly higher in the older group (compared to 66–74 or 70–74) [4, 18, 21, 25], whilst one study showed a slight decrease in 10-year cumulative incidence of invasive breast cancer among older women [23]. One study compared women who continue screening with women who stop screening [50] and demonstrated a significant increase in localised invasive breast cancer incidence in those who continue screening (RD 1.65 (95%CI 1.21–2.03)).

Of the seven studies that reported on DCIS, four showed either no change or a decrease in DCIS rates as women aged (1.6 v 2.0 per 1000 screens [4], 2.6 vs 2.6 per 1000 [18, 21], 10 year cumulative incidence 1% v 0.7% [23]) and two showed an increase (rate per 1000 women screened 70–74years: 0.87 vs 75+ years: 0.97 [25], RR compared to no screening 4.9 (3.5–6.9)) [42]. Richman [50] showed an increased risk in DCIS detection in women who continue screening compared to women who stop screening (RD 0.64 (95% CI 0.46-0.79)).

### Breast cancer characteristics

Cancer characteristics that were presented included proportion of cancers that were node positive; stage distribution of the cancers detected; and tumour size (Table 5).

In the three studies that compared younger and older women, older women were less likely to have an advanced stage cancer detected [21, 25, 38] however the comparators included some age-groups as young as 40-49 years. These findings are consistent with studies reporting smaller tumour sizes as women age [25, 38], and with regular repeat screening interval [46].

### Treatment patterns

Treatment patterns were reported by three studies [24, 26, 27] (Table 6) however only one study included a comparator [26]. Almost all women 75 years and older with screen-detected cancers received some form of treatment. In the one study with a comparison there was evidence that continuing to screen was associated with a higher proportion of less radical surgical treatment, (radical mastectomy: continuing screening vs stopping screening: 14.2% v 17.0%) [26].

## Discussion

The studies included in our systematic review used heterogeneous methods to assess and report on a range of outcomes for mammography screening in older women. Given this heterogeneity, we have summarised study-specific findings in evidence tables (since pooling of data would not be appropriate), noting there was mixed evidence about the benefits of continuing mammography screening beyond the age of 75 years. The few studies that reported on breast cancer mortality as outcome gave contrasting (mixed) results: about equal numbers of studies showed a beneficial effect [32–35, 39–41] or no effect on mortality [23, 26, 30, 37, 44, 45, 50] and/or used un-informative comparisons.

Although fewer studies reported on the harms of screening beyond the age of 75, evidence presented on various harms (false positives, recalls, biopsy, and overdiagnosis) were generally more consistent. Specifically, there was consistent evidence that screening into older age increases overdiagnosis [19, 30, 31, 39, 47, 49, 50] which can be partially explained due to the shorter follow-up time possible with older women, and higher competing causes of mortality [51].

The evidence reported in this review should be interpreted factoring in the various limitations we identified. Many studies used comparisons that were not informative about the health impact of screening into older age or used comparisons that could bias towards an effect from screening: the ideal comparison to assess the impact of screening beyond the age of 75 would be to compare those who continue screening with those who stop screening at the age of 75. Only two observational studies undertook this comparison [26, 50], although some modelling studies simulated this scenario [30, 34, 39, 47]. The results of these studies are summarised in Appendix 2. When considering only these studies three of the modelling studies indicated a benefit, whereas both observational studies and one modelling study did not, and all six studies reported harms. One study showed similar estimates in breast cancer deaths averted [30], two showed no difference in risk of breast cancer death [26, 50], two indicated benefit in terms of life years gained [34, 47] and two estimated a reduction in breast cancer mortality [34, 39]. We also see an additional number of false positive screens [39], an increase in the false positive biopsy rate [39], an increase in the incidence of breast cancer [50] (including increase in both invasive cancer detection rates [50] and DCIS rates [50])and an increase in overdiagnosis [30, 39, 47, 50].

The other comparisons made by included studies do not provide direct evidence on the health benefits and harms of continuing to screen beyond age 75. Studies that compare older women to younger age groups, compare according to screening history or screening interval, or compare by whether a cancer is screen-detected or diagnosed clinically (by physician or patient) are prone to lead time bias and as a result may be inherently biased towards screening, and do not tell us how health outcomes change if the woman chooses to stop vs continue screening beyond age 75. For example, a study with a more informative comparison (stop vs continue screening) [26] indicates a non-significant breast cancer death hazard ratio (1.00; 95% CI 0.83–1.19), whereas a study with a less informative comparison (within age-group clinical detection vs screen-detected) [32] suggests a significant benefit to screen detection (HR 0.5; 95% CI 0.31–0.82). As such, the results of many of the studies with less informative comparisons need to be interpreted with caution.

Several studies reported screening detection measures, showing similar or higher cancer detection rates (depending on the comparison used) or PPV for recall (detection yield) for screening older women [4, 18, 21, 26, 43] although several studies did not have a comparator [22, 24, 27]. These metrics provide information about the performance of the screening process, but they provide less knowledge about the health benefit of continuing to screen. This comparison is particularly problematic when being made by age-groups as the detection metrics generally differ between younger and older women. For example, mammograms are more sensitive in older women, and underlying cancer rates higher, so more cancer detection would be expected at screening in older women (compared to younger groups) but this is not equivalent to evidence on whether screening beyond 75 years and older as opposed to stopping confers a mortality benefit.

Likewise, comparing cancer characteristics and treatment patterns between younger and older women is un-informative about screening effects because breast cancer biology and treatment are known to differ between younger and older women, so in extreme age, i.e. 75 years and older, these differences would be expected and could be more evident. Therefore reported differences related to cancer characteristics between age-groups do not provide direct evidence about health benefit of screening into older age.

It is important to note that the potential benefits of screening do change as women age. The sensitivity of screening increases, but so too does the competing risk of death from other causes. Screening older women might not be effective in terms of mortality reduction, even if mammography screening detects early-stage breast cancer well, if most deaths in those older than 75 are not from breast cancer. This is highlighted in the data from Demb [23], where the cumulative incidence of breast cancer deaths is around 0.3% and so screening older women will make only a small difference to this proportion. At the same time, cumulative incidence of other causes of death becomes very dominant and increases significantly as women age (66–74 years: 14.5% (95% CI: 14.3–14.8%); 75–84 years: 35.7% (95% CI:35.3–36.1%); 85 years +: 65.4% (95% CI: 64.3–66.5%), also increasing with higher co-morbidities [23]. Considering co-morbidities, one study highlighted that the estimated breast cancer mortality reduction from screening decreased with increasing age and with higher co-morbidity score [39], and other studies reported that incremental life-years gained for continuing screening diminished in those with more severe co-morbidity [31, 39]. On the other hand, one could argue that early-detection of breast cancer in this age-group may reduce treatment burden, and hence represents an important outcome (even if limited evidence on mortality reduction). Very few eligible studies reported treatment patterns and only one had a comparison, highlighting that those who continued to screen were more likely to receive conservative breast surgery than those who stopped screening beyond 75 years [26].

Quality appraisal showed all studies were prone to bias with most observational studies rated as serious risk of bias, and most modelling studies rated at a moderate risk of bias. However, caution should be taken when interpreting these findings because we applied established quality appraisal tools for observational studies [13, 14], and in the absence of a standard tool for appraising modelling studies we adapted criteria from Carter et al. [15]. As a result, it seems likely that more stringent criteria were applied to observational studies. This highlights the challenges in assessing the quality of modelling studies, especially with regards to the assumptions applied in models. For example, when modelling overdiagnosis, only three [31, 39, 47] of the five papers included assumptions allowing for DCIS that is non-progressive, and only two studies [39, 47] included non-progressive invasive cancer in their assumptions. Many of the modelling papers did not state this clearly in their methods, and further details were sought from cited earlier work. It is possible that the assumptions made in these studies may not be consistent with current understanding of the natural history of breast cancers with regards to non-progressive disease, which could bias estimates resulting in an under-estimation of overdiagnosis.

As with all studies evaluating the impact of screening programmes, lead time bias must be considered. The studies included in this review are no exception: with no RCTs available for inclusion, lead time bias will be evident in included studies. As such, the benefit shown in survival of all four studies cannot be taken as evidence of screening benefit, as their results will be affected by lead time bias [32, 35, 36, 49].

Given the limited quality and mixed evidence about the benefits of continuing mammography screening beyond the age of 75 years, older women should be presented with the opportunity to make an informed decision based on their values and an understanding of the lack of evidence in this area. Decision aids have been shown as effective in enabling older women to make more informed decisions regarding mammography screening [52].

## Conclusion

Despite many studies having reported on outcomes of screening women aged 75 and older, findings from this systematic review highlight the limited evidence available from high quality studies to make a recommendation *for or against* continuing breast screening beyond the age of 75 years. Many of the comparisons used in published studies are not directly informative as far as benefit or harms associated with continuing to screen (as opposed to stopping) beyond 75. Further studies with more informative comparisons, specifically comparing continuing versus stopping screening at 75 years, are required before definitive recommendations can be made.

### Supplementary information

Supplementary Appendices 1 and 2

## Data Availability

Any requests for data can be made by emailing the corresponding author.
