# Supplementary material for: Health benefits and harms of mammography screening in older women (75+ years)—a systematic review
Source: Br J Cancer. 2023 Nov 29;130(2):275–96. doi: 10.1038/s41416-023-02504-7 (PMC10803784; doi:10.1038/s41416-023-02504-7)
Supplement: Supplementary file 1 — Supplementary Appendices 1 and 2 [file 41416_2023_2504_MOESM1_ESM.docx]

| **Section and Topic** | **Item #** | **Checklist item** | **Location where item is reported** |
| --- | --- | --- | --- |
| **TITLE** | | |  |
| Title | 1 | Identify the report as a systematic review. | 1 |
| **ABSTRACT** | | |  |
| Abstract | 2 | See the PRISMA 2020 for Abstracts checklist. | 2 |
| **INTRODUCTION** | | |  |
| Rationale | 3 | Describe the rationale for the review in the context of existing knowledge. | 3 |
| Objectives | 4 | Provide an explicit statement of the objective(s) or question(s) the review addresses. | 3 |
| **METHODS** | | |  |
| Eligibility criteria | 5 | Specify the inclusion and exclusion criteria for the review and how studies were grouped for the syntheses. | 4 |
| Information sources | 6 | Specify all databases, registers, websites, organisations, reference lists and other sources searched or consulted to identify studies. Specify the date when each source was last searched or consulted. | 4 |
| Search strategy | 7 | Present the full search strategies for all databases, registers and websites, including any filters and limits used. | Appendix 1 |
| Selection process | 8 | Specify the methods used to decide whether a study met the inclusion criteria of the review, including how many reviewers screened each record and each report retrieved, whether they worked independently, and if applicable, details of automation tools used in the process. | 4 |
| Data collection process | 9 | Specify the methods used to collect data from reports, including how many reviewers collected data from each report, whether they worked independently, any processes for obtaining or confirming data from study investigators, and if applicable, details of automation tools used in the process. | 5 |
| Data items | 10a | List and define all outcomes for which data were sought. Specify whether all results that were compatible with each outcome domain in each study were sought (e.g. for all measures, time points, analyses), and if not, the methods used to decide which results to collect. | 4-6 |
|  | 10b | List and define all other variables for which data were sought (e.g. participant and intervention characteristics, funding sources). Describe any assumptions made about any missing or unclear information. | 4-5 |
| Study risk of bias assessment | 11 | Specify the methods used to assess risk of bias in the included studies, including details of the tool(s) used, how many reviewers assessed each study and whether they worked independently, and if applicable, details of automation tools used in the process. | 5 |
| Effect measures | 12 | Specify for each outcome the effect measure(s) (e.g. risk ratio, mean difference) used in the synthesis or presentation of results. | 4 |
| Synthesis methods | 13a | Describe the processes used to decide which studies were eligible for each synthesis (e.g. tabulating the study intervention characteristics and comparing against the planned groups for each synthesis (item #5)). | 5 |
|  | 13b | Describe any methods required to prepare the data for presentation or synthesis, such as handling of missing summary statistics, or data conversions. | N/A |
|  | 13c | Describe any methods used to tabulate or visually display results of individual studies and syntheses. | 5 |
|  | 13d | Describe any methods used to synthesize results and provide a rationale for the choice(s). If meta-analysis was performed, describe the model(s), method(s) to identify the presence and extent of statistical heterogeneity, and software package(s) used. | 5 |
|  | 13e | Describe any methods used to explore possible causes of heterogeneity among study results (e.g. subgroup analysis, meta-regression). | N/A |
|  | 13f | Describe any sensitivity analyses conducted to assess robustness of the synthesized results. | N/A |
| Reporting bias assessment | 14 | Describe any methods used to assess risk of bias due to missing results in a synthesis (arising from reporting biases). | N/A |
| Certainty assessment | 15 | Describe any methods used to assess certainty (or confidence) in the body of evidence for an outcome. | N/A |
| **RESULTS** | | |  |
| Study selection | 16a | Describe the results of the search and selection process, from the number of records identified in the search to the number of studies included in the review, ideally using a flow diagram. | 5 and Figure 1 |
|  | 16b | Cite studies that might appear to meet the inclusion criteria, but which were excluded, and explain why they were excluded. | 5, Table 1 |
| Study characteristics | 17 | Cite each included study and present its characteristics. | 5-8, Table 1 |
| Risk of bias in studies | 18 | Present assessments of risk of bias for each included study. | 5-6, Table 1, Online appendix 2 |
| Results of individual studies | 19 | For all outcomes, present, for each study: (a) summary statistics for each group (where appropriate) and (b) an effect estimate and its precision (e.g. confidence/credible interval), ideally using structured tables or plots. | 5-8, Tables 1 -6 |
| Results of syntheses | 20a | For each synthesis, briefly summarise the characteristics and risk of bias among contributing studies. | 5-8 |
|  | 20b | Present results of all statistical syntheses conducted. If meta-analysis was done, present for each the summary estimate and its precision (e.g. confidence/credible interval) and measures of statistical heterogeneity. If comparing groups, describe the direction of the effect. | NA |
|  | 20c | Present results of all investigations of possible causes of heterogeneity among study results. | NA |
|  | 20d | Present results of all sensitivity analyses conducted to assess the robustness of the synthesized results. | 8 Online appendix 2 |
| Reporting biases | 21 | Present assessments of risk of bias due to missing results (arising from reporting biases) for each synthesis assessed. | NA |
| Certainty of evidence | 22 | Present assessments of certainty (or confidence) in the body of evidence for each outcome assessed. | NA |
| **DISCUSSION** | | |  |
| Discussion | 23a | Provide a general interpretation of the results in the context of other evidence. | 8-10 |
|  | 23b | Discuss any limitations of the evidence included in the review. | 8-10 |
|  | 23c | Discuss any limitations of the review processes used. | 8-10 |
|  | 23d | Discuss implications of the results for practice, policy, and future research. | 10 |
| **OTHER INFORMATION** | | |  |
| Registration and protocol | 24a | Provide registration information for the review, including register name and registration number, or state that the review was not registered. | 5 |
|  | 24b | Indicate where the review protocol can be accessed, or state that a protocol was not prepared. | 5 |
|  | 24c | Describe and explain any amendments to information provided at registration or in the protocol. | NA |
| Support | 25 | Describe sources of financial or non-financial support for the review, and the role of the funders or sponsors in the review. | 1 |
| Competing interests | 26 | Declare any competing interests of review authors. | 1 |
| Availability of data, code and other materials | 27 | Report which of the following are publicly available and where they can be found: template data collection forms; data extracted from included studies; data used for all analyses; analytic code; any other materials used in the review. | NA |

*From:*  Page MJ, McKenzie JE, Bossuyt PM, Boutron I, Hoffmann TC, Mulrow CD, et al. The PRISMA 2020 statement: an updated guideline for reporting systematic reviews. BMJ 2021;372:n71. doi: 10.1136/bmj.n71

For more information, visit: <http://www.prisma-statement.org/>

| Online Appendix 2: Modelling studies assumptions on progressive disease and risk of bias rating | | | | | | | | | | | |
| --- | --- | --- | --- | --- | --- | --- | --- | --- | --- | --- | --- |
| **Study reference** | **type of model** | **model name** | **allow for non-progressing DCIS in assumptions** | | **allow for non progressing invasive** | | **Other comments** | **assumption rating** | **validation** | **validation rating** | **Overall RoB** |
|  |  |  | **Yes/No/Unknown** | **What assumptions are used** | **Yes/No/Unknown** | **What assumptions are used and what are they based on?)** |  | **good/fair/poor/ not reported** | **validated using different population data** |  | **high/ moderate/low** |
| Arleo 2017 | Four models are microsimulations (Model E, Model M, Model G-E, and Model W), Model D uses an analytic approach, and Model S is a hybrid analytic/microsimulation.  https://resources.cisnet.cancer.gov/registry/site-summary/breast/ | 6 models from CISNET | Unknown from this paper, but according to Mandelblatt 2015 technical report: Yes for model D (post 2009), E, G-E, W, No for models M and S |  | Unknown from this paper, but according to Mandelblatt 2015 technical report: Yes for model W, No for models D, E, G-E, M, S | Two CISNET models make the assumption that invasive breast cancers may fail to progress; however, extremely few cases of nonprogressive invasive breast cancer have been reported in the literature. | We used the 2009 and 2015-2016 CISNET breast cancer–specific models  the decision was made not to include overdiagnosis in this study’s risk assessment. However, unless breast cancers actually can regress and disappear (which no one has ever observed for an invasive cancer found by mammography), delaying the age at which screening is started and extending the time between screens will have no effect on “overdiagnosis”, since “overdiagnosed” cancers still will be present at the age of 45 or 50 and still will be present whether screening is annual or biennial. | Good | As per reference paper, several data sources were used in the validation | Yes | Low |
| Boer 1995 | Microsimulation | Microsimulation screening analysis-(MISCAN) | No | the disease model is based on a three-stage division of the development of invasive breast cancer in which the stage reflects the tumour size. A proportion of the invasive breast cancer is preceded by a screen detectable DCIS. The screen detectable stages have an exponentially distributed sojourn time with an age-dependent mean. | No |  | From reference paper: "It is assumed that all screen-detectable DCIS cases are progressive, ie., they eventually develop into an invasive form of breast cancer. This assumption cannot be justified on the basis of available data. One could just as readily assume that some fraction of DCIS cases are nonprogressive. The age-specific incidence of the preclinical stage is obtained by shifting the clinical incidence rate to younger ages, by a number of years corresponding to the average (age-specific) total duration of the preclinical stages. This implies 100% progression of preclinical disease and excludes overdiagnosis by screening (except for inevitable cases in which women die from other independent causes in the period between screen detection and clinical diagnosis without screening). This assumption is in agreement with findings from the randomized trial" | Poor | No mention of validation | No | High |
| Jansen 1997a | Simulation | Model for evaluation of breast cancer screening (MBS) | No | Simulates individual mammary tumors based on the selection from appropriate distributions of characteristic parameters such as age at tumor onset, growth rate, lifetime expectancy and tumour diameter at detection du to screening and at spontaneous discovery the survival is calculated according to the data of Tabar et al | No |  | As per reference paper above. Although tumour growth rate is built into the model, it is not noted how the growth rate is calculated other than the paragraph above - implies that all tumours are progressive | Fair | No mention of validation | No | Moderate |
| Jansen 1997b | Simulation | Model for evaluation of breast cancer screening (MBS) | No |  | No |  | From reference paper: it is assumed in MBS that, starting at a randomly selected onset age, a tumour grows exponentially, according to a selected tumour volume doubling time. At onset MBS assigns to the tumour a size of 2048 cells (equivalent to 11 cell divisions starting from a single cell) | Fair | No mention of validation - only use Swedish female population data | No | Moderate |
| Kregting 2021 | Microsimulation | Microsimulation screening analysis-breast model (MISCAN-Breast) | No | In the model, BC starts with a pre-clinical DCIS that can progress to invasive stages T1A, T1B, T1C and T2+, respectively. A tumour can become screen detected, clinically detected or can progress to the next preclinical stage. | No |  | Previously calibrated for the natural history of breast cancer and breast cancer survival rates with data up to 2015 (see ref 14) | Fair | No mention of validation | No | Moderate |
| Lansdorp-vogelaar 2014 | Microsimulation | Microsimulation screening analysis-Fatal diameter (MISCAN-Fadia) and Georgetown-Einstein (G-E Model) | Yes | MISCAN-Fadia model - as per reference paper- Percentage of regressive DCIS ranges from around 1% for women over 75yr.  G-E model - UK | UK | No mention in paper or reference paper. | MISCAN-Fadia includes a DCIS sub model, taken without modification from the standard MISCAN breast cancer model ( 7 , 8 ) and based on data from the screening trials in Utrecht and Nijmegen (The Netherlands). In this submodel, there are three different types of preclinical DCIS: regressive DCIS, DCIS that will be diagnosed clinically, and DCIS that will progress to invasive disease. All have a mean duration of 5.22 years. The distribution between the three types depends on age (see Table 6 ). For screening of preclinical DCIS, the standard MISCAN value of 0.4 for the sensitivity of DCIS is used for 1975 and is assumed to increase linearly to 0.8 in 2000. Both screen-detected DCIS and clinically detected DCIS are assumed to have a 100% survival. | Fair | In reference papers | Yes | Moderate |
| Mandelblatt 2009 | Four models are microsimulations (Model E, Model M, Model G, and Model W), Model D uses an analytic approach, and Model S is a hybrid analytic/microsimulation.  https://resources.cisnet.cancer.gov/registry/site-summary/breast/ | 6 models from CISNET: Model D, Model E, Model G, Model M, Model S, Model W | Yes for models E and W, No for models D, G, M, S.  *Models S and D include only invasive cancer.* | Models E and W specifically assume that some portions of DCIS are nonprogressive and do not result in death. | Yes for model W, No for models D, E, G, M, S | Model W also assumes that some cases of small invasive cancer are nonprogressive. | This study uses 6 different models developed independently within CISNET network. Each model has a different structure and assumptions and some varying input variables.  We define overdiagnosis as the proportion of cases in each strategy that would not have clinically surfaced in a woman’s lifetime (because of lack of progressive potential or death from another cause) among all cases arising from age 40 years onward. The absolute estimate of overdiagnosis varied between models depending on whether DCIS was or was not included and on the assumptions related to progression of DCIS and invasive disease. | Fair | In previous papers | Yes | Moderate |
| Schousboe 2022 | Markov microsimulation | Markov | Yes | If a woman develops DCIS, she can remain in that state  We considered the excess cases of DCIS found when screening mammography is extended 5 years to be cases of overdiagnosis. | Yes, in secondary analyses | We did secondary analyses assuming that 5% of invasive breast cancer cases are also instances of overdiagnosis (i.e. localized lesions that never progress to more advanced stages). |  | Fair | Explicit statement noting this | Yes | Moderate |
| van Ravesteyn 2015 | Microsimulation | 3 models from CISNET: Model E, Model G-E, Model W | Yes | All 3 models included DCIS with 3 different types of preclinical DCIS: regressive DCIS, DCIS that is diagnosed clinically, and DCIS that progresses to invasive disease | Yes for model W, No for models E and G-E | Model W also assumed that some cases of small invasive cancer are nonprogressive and have limited malignant potential (LMP). | The models used incidence and mortality data by age and calendar year (1975–1999) from the Surveillance, Epidemiology, and End Results (SEER) Program to estimate natural history parameters, including the transition rates of DCIS becoming invasive or clinically diagnosed and DCIS regression rates (model E) and the proportion of DCIS with LMP (model G-E and model W). | Good | Explicit statement noting this has been done in previous studies | Yes | Low |

CISNET = Cancer Intervention and Surveillance Modeling Network

RoB = risk of bias
